# The Skin Microbiome: A Focus on Pathogens and Their Association with Skin Disease

**DOI:** 10.1371/journal.ppat.1004436

**Published:** 2014-11-13

**Authors:** Keisha Findley, Elizabeth A. Grice

**Affiliations:** 1 Social and Behavioral Research Branch, National Human Genome Research Institute, Bethesda, Maryland, United States of America; 2 Department of Dermatology, University of Pennsylvania, Perelman School of Medicine, Philadelphia, Pennsylvania, United States of America; University of North Carolina at Chapel Hill School of Medicine, United States of America

## What Is the Human Microbiome and Why Is It Important?

Microbes are present in every environmental niche and live in close association with humans and each other. Members of these communities include bacteria, archaea, viruses, fungi, and protists. In the environment, microbes perform various roles and are essential in agriculture and food production. In humans and animals, microbes provide protection against foreign invaders, educate and stimulate the immune response, produce antimicrobials, aid in digestion, and produce vitamins, among a host of other functions.

In the last decade, researchers have actively investigated the impact of microbes and their gene products in human health and disease. This field of research is defined as the human microbiome, which is the totality of the microbes and their genes in and on the human body. To better understand the impact of microbes on health and disease, multiple body sites have been investigated, including skin, lungs, the nasal passage, the oral cavity, and the gastrointestinal and urogenital tracts. The results of these studies have revealed that each body site is home to a unique microbial community. Shifts in microbial communities can result from multiple factors, including environment, genetic variation, lifestyle and hygienic factors, and the immune system, and have been associated with many diseases.

## What Is the Difference between Culture-Dependent and –Independent Methods of Microbial Community Characterization?

Up until the 1980s, microbiologists routinely relied on culture-dependent methods for microbial isolation, identification, and characterization. Colony morphology, stains (i.e., Gram stain), biochemical characteristics (i.e., coagulase test), motility tests, antibiotic resistance profiles, and other characteristics guided bacterial and/or fungal identification and taxonomy. However, this approach has several limitations, including an inability to mimic in vivo conditions and selection against slow-growing and/or fastidious organisms. With recent advances in sequencing technologies and development of bioinformatics tools and reference databases, researchers are now better equipped to capture microbial diversity without the biases of culture-based approaches.

Culture-independent methods of microbial identification rely on a targeted amplicon strategy, which employs highly conserved microbe-specific molecular markers and does not rely on growing isolates in pure culture. The 16S ribosomal RNA (rRNA) gene is used for bacterial identification, while fungi and other microeukaryotes are identified using either the 18S rRNA gene or the Internal Transcribed Spacer (ITS) region. A complementary approach to amplicon-based surveys is whole genome shotgun metagenomics. With this approach, one can identify the microbiota present and gain insight into the functional potential of the microbiota in an untargeted manner.

## What Is the Diversity of the Human Skin Microbiota, as Revealed by Culture-Independent Methods?

The skin is our first line of defense against foreign invaders and is also home to a diverse population of microbes. The majority of these microbes are commensals (nonpathogenic permanent residents) or transients (temporary residents) of the human microbiota. In pathogenic interactions, only the microbe benefits, while the host is eventually harmed. Many skin pathogens can be typically found living on the skin as commensals, but microbial dysbiosis (or microbial imbalance), host genetic variation, and immune status may drive the transition from commensal to pathogen.

Analysis of bacterial diversity on human skin employing 16S rRNA sequencing revealed that multiple skin sites exhibited greater bacterial diversity than in the gut and oral cavity; interpersonal variation varied significantly within the population studied, and the temporal stability of the analyzed skin microbial communities remained relatively stable [1]. Physiological characteristics of various skin sites are associated with different levels of bacterial diversity [2]. Spatially, the skin microbiota may extend to subepidermal compartments [3]. Findley et al. characterized fungi on healthy human skin using sequencing of the ITS region and showed the greatest diversity of fungi was found on the feet, with intermediate levels of diversity on the hand and forearm [4]. The core body sites were less diverse but more stable over time and were primarily colonized by the most common skin commensal, *Malassezia*. Additionally, using shotgun metagenomic sequencing techniques, several viruses have been reported in association with healthy skin and skin disorders, including human papillomavirus, human polyomaviruses, circoviruses, and bacteriophages [5], [6].

## What Are Some Key Pathogenic Skin Microbes, the Diseases They Cause, and Their Relationship to the Skin Microbiome?

In the following sections we highlight key findings that implicate specific microbes in skin disease, but whose pathogenesis may be complicated by microbial community interactions and/or host-microbe interactions. The specific microbes discussed include *Staphylococcus aureus*, *Propionibacterium acnes*, and *Malassezia* spp., all of which are known skin commensals but also exhibit pathogenic potential under certain conditions. There are other well-characterized skin pathogens that have been definitively linked to dermatological disorders, but will not be examined in depth here. These include, but are not limited to, the dermatophyte *Trichophyton* (causing onychomycosis and tinea pedis), *Corynebacterium minutissimum* (causing erythrasma), papillomaviruses (causing warts), *Candida* fungal species (causing cutaneous candidiasis and diaper rash), and *Pseudomonas aeruginosa* (implicated in green nail syndrome, gram-negative folliculitis, and toe web infections).

### *Staphylococcus aureus*

*Staphylococcus aureus* is a gram-positive bacteria and facultative anaerobe. In some cases, it may be a skin commensal and colonizes the nares in approximately 20% of the population [7]. However, *S. aureus* is a major cause of skin and soft tissue infections (SSTI) (Table 1). The sequenced genome of *S. aureus* has revealed multiple virulence factors encoded by phages, plasmids, and pathogenicity islands [8]. In order to evade detection by the host's immune system, *S. aureus* produces a variety of enzymes and toxins to successfully establish infection [8].

**Table 1:** Skin microbes discussed and their associated skin diseases.

Genus	Species	Primary Host	Associated Skin Disease(s)
**Bacteria**			
*Staphylococcus*	*aureus*	human nares	atopic dermatitis, skin and soft tissue infections
*Propionibacterium*	*acnes*	human skin	acne, dandruff, psoriasis, blepharitis
**Fungi**			
*Malassezia*	*dermatis*, *furfur*, *globosa*, *japonica*,*obtusa*, *restricta*, *slooffiae*, *sympodialis*, *yamatoensis*	human skin	atopic dermatitis, tinea versicolor, psoriasis

*S. aureus* colonization and infection are associated with atopic dermatitis (AD), a chronic and highly inflammatory skin disease in children and adults (Table 1). Kong et al. recently showed, using 16S rRNA sequencing, that in a pediatric population of AD patients, temporal shifts in the skin microbiota occur over three disease stages, baseline, flare (active disease), and post flare (after treatment has been administered), as compared to healthy age-matched controls. In particular, lesional skin bacterial diversity decreased during the flare stage, parallel with increased relative abundance of *S. aureus*, but increased during the post flare status, indicative of a link between disease severity and microbial diversity [9]. An explanation for the observed increase in *S. aureus* in lesional skin could in part be due to a reduction in the host's ability to produce antimicrobial peptides, which normally prevent invasion by *S. aureus* [10].

### *Propionibacterium acnes*

*Propionibacterium acnes* is a gram-positive, oxygen tolerant anaerobe, and one of the most common skin commensal bacteria. *P. acnes* resides in hair follicles and sebaceous glands where it metabolizes sebum triglycerides to release free fatty acids. *P. acnes* on skin may inhibit invasion by pathogenic microbes like *S. aureu*s and *Streptococcus pyogenes* through the production of these short-chain fatty acids [11]. *P. acnes* also produces propionic acid and secretes bacteriocins such as thiopeptide, which suppress the growth of *S. aureus* and other pathogenic microbes [12], [13].

*P. acnes* is associated with the common adolescent skin condition acne vulgaris (Table 1) [14], though the disease is likely multifactorial with contributions from the immune system, genetics, and the environment. The colonization of skin with *P. acnes* is age-dependent [15], increasing in parallel with maturation of sebaceous glands during puberty. Sequencing of the bacterial rRNA gene indicates that certain strains of *P. acnes* may be associated with acne and contain unique genetic elements that contribute to their virulence [11], [16]. Probiotic applications may offer some benefit for treatment, as *P. acnes* growth is inhibited by succinic acid, a fatty acid fermentation product of the commensal *Staphylococcus epidermidis* [17]. Acne may also be an ideal candidate for bacteriophage therapy, as *P. acnes* bacteriophage are limited in genetic diversity, have a broad host range, and are unable to form stable lysogens within their hosts [18].

### *Malassezia*

*Malassezia* is a genus of dimorphic and primarily lipophilic fungi formerly known as *Pitysporum*. It is the most abundant fungal skin commensal, representing 50%–80% of total skin fungi, most common in oily areas such as the face, scalp, and back. *Malassezia* species live in the infundibulum of the sebaceous glands where they feed on lipids found in human sebum [19]. *Malassezia* has been hypothesized as the infectious agent in several skin disorders, namely dandruff, atopic dermatitis, tinea versicolor, and to a lesser extent psoriasis (Table 1) [20]. *Malassezia* produces lipases, phospholipases, and allergens (*Mala* genes), which can damage the integrity of the skin by inducing inflammation and triggering an immune response [21].

Dandruff is a common skin disorder marked by abnormal flaking and itching of the scalp. Antifungal agents are quite effective in treatment, thus suggesting a fungal component to the disease. Free fatty acids such as oleic acid produced by *Malassezia* lipases may be responsible for altering skin barrier permeability, leading to irritation and inflammation of the scalp in predisposed individuals [22]. A recent culture-independent study on French subjects with and without dandruff suggested that disequilibrium between bacteria and fungi, including *Malassezia spp.*, on the scalp is associated with this condition [23].

## Exciting New Directions in Skin Microbiome Research

The skin microbiome in healthy adults has been defined using culture-independent methods, and changes in microbial populations associated with disease have been identified. Future efforts will need to focus on proving causation and functional relevance of shifts in microbial populations that are associated with certain conditions, such as those described here. It is clear that many factors other than the microbiome underlie transitions between health and disease states, including the immune system, the environment, and genetic variation. Recent findings suggest a complex interplay between our cutaneous immune system and the microbiome. For example, skin commensals were shown to control the cutaneous inflammatory milieu and tune resident T cell function [24]. The skin commensal *S. epidermidis* has also been shown to inhibit inflammation via pattern recognition receptor–mediated cross talk [25]. On the other hand, cutaneous immunity has been shown to impact microbial communities [26]. Additional exciting new directions may focus on the identification of novel treatments and diagnostic and prognostic tools that leverage our knowledge of skin microbial communities.
